# Perioperative Extracorporeal Membrane Oxygenation Support for Acute Respiratory Distress Syndrome Aggravated by Hepatopulmonary Syndrome in Deceased Donor Liver Transplantation: A Case Report

**DOI:** 10.3390/medicina59081422

**Published:** 2023-08-04

**Authors:** So Ron Choi, Seung Cheol Lee, Tae Young Lee, Ji Wook Jung, Min A Kim, Sang Yoong Park

**Affiliations:** Department of Anesthesiology and Pain Medicine, Dong-A University Hospital, 26 Daesingongwon-ro, Busan 49201, Republic of Korea; choisr@dau.ac.kr (S.R.C.); k57501@dau.ac.kr (S.C.L.); pinpd@dau.ac.kr (T.Y.L.); wnr2749@gmail.com (J.W.J.); kma4477@gmail.com (M.A.K.)

**Keywords:** acute liver failure, acute respiratory distress syndrome, deceased donor liver transplantation, hypoxemia, veno-venous extracorporeal membrane oxygenation

## Abstract

*Background*: Extracorporeal membrane oxygenation (ECMO) is an accommodation of the cardiopulmonary bypass technique that can support gas exchange and hemodynamic stability. It is used as a salvage maneuver in patients with life-threatening respiratory or cardiac failure that does not respond to conventional treatment. There are few case reports of successful perioperative use of ECMO, especially preoperatively, in liver transplantation (LT). Here, we report an experience of successful anesthetic management in deceased donor liver transplantation (DDLT) by applying perioperative veno-venous (VV) ECMO support in the setting of acute respiratory distress syndrome (ARDS) aggravated by hepatopulmonary syndrome (HPS). *Case*: A 25-year-old female (156.0 cm, 65.0 kg), without any underlying disease, was referred to our emergency department for decreased mentality. Based on imaging and laboratory tests, she was diagnosed with acute liver failure of unknown cause combined with severe ARDS aggravated by HPS. Since the patient faced life-threatening hypoxemia with a failure of conventional ventilation maneuvers, preoperative VV ECMO was initiated and maintained during the operation. The patient remained hemodynamically stable throughout DDLT, and ARDS showed gradual improvement after the administration of VV ECMO. As ARDS improved, the patient’s condition alleviated, and VV ECMO was weaned on postoperative day 6. *Conclusions*: This case demonstrates that VV ECMO may be a useful therapeutic option not only during the intraoperative and postoperative periods but also in the preoperative period for patients with liver failure combined with reversible respiratory failure.

## 1. Introduction

Extracorporeal membrane oxygenation (ECMO) is an accommodation of the cardiopulmonary bypass technique that can support gas exchange and hemodynamic stability. It is used as a salvage maneuver in patients with life-threatening respiratory or cardiac failure that does not respond to conventional treatment [1,2]. While the role of ECMO and its use are widely known in the non-transplantation literature, there are few case reports of successful perioperative use of ECMO, especially preoperatively, in liver transplantation (LT) [3,4].

Acute respiratory distress syndrome (ARDS) is a clinical syndrome that represents rapidly progressing non-cardiac hypoxemia and is relatively common in patients undergoing liver transplantation [5]. Its associated mortality remains high, and given the limited therapeutic options for ARDS, treatment focuses on providing adequate gas exchange through conventional ventilation maneuvers [6]. Veno-venous (VV) ECMO has emerged as an alternative treatment to conventional therapeutic interventions, allowing support for potentially reversible ARDS [1]. For patients experiencing respiratory failure while waiting for LT, VV ECMO may be a feasible choice if it is appropriate for their condition. Here we report an experience of successful anesthetic management in deceased donor liver transplantation (DDLT) by applying perioperative VV ECMO support in the setting of ARDS aggravated by hepatopulmonary syndrome (HPS).

## 2. Case Description

A 25-year-old female (156.0 cm, 65.0 kg) without any underlying disease was referred to our emergency department for decreased mentality. The previous day, she was admitted to our institution with epigastric pain and received esomeprazole and cimetropium bromide. An allergic reaction was observed after administration, so the infusion was discontinued and peniramin was administered intravenously with fluid hydration. She was discharged once her symptoms subsided. The following day, she was found in a stuporous state and was transported to our emergency department by ambulance. Imaging and laboratory tests were conducted, ruling out brain injury and drug intoxication. Initial laboratory results revealed an aspartate aminotransferase level of 1398 U/L, alanine aminotransferase level of 5172 U/L, total bilirubin/direct bilirubin level of 8.7/5.2 mg/dL, pH of 7.357, ammonia level of 380 umol/L, lactate level of 62.9 mg/dL, serum creatinine level of 2.34 mg/dL, and international normalized ratio (INR) of 4.56. The patient’s initial vital signs were 68/40 mmHg for blood pressure, 134 beats/min for heart rate, and 90% for oxygen saturation. Due to an irregular breathing pattern and hypotension, inotropics were administered, and tracheal intubation with mechanical ventilation support was performed. The patient was registered for DDLT under the diagnosis of acute liver failure of unknown cause and was admitted to the intensive care unit (ICU) for management with vasopressors and mechanical ventilation.

**Figure 1:**
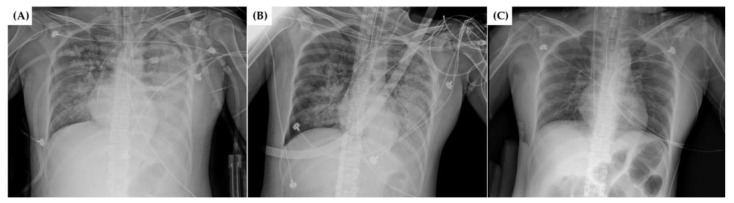
Chest radiography (**A**) before ECMO, (**B**) after ECMO, and (**C**) after ECMO weaned. ECMO: extracorporeal membrane oxygenation.

After admission to the ICU, the patient’s condition deteriorated rapidly, presenting with metabolic acidosis (pH < 7.300), lactic acidosis (lactate > 100 mg/dL), azotemia (serum creatinine > 2.0 mg/dL), increased demand for vasopressors, and poor oxygenation. Continuous renal replacement therapy (CRRT) was administered via a hemodialysis catheter cannulated at the left femoral vein. The fraction of inspired oxygen (FiO_2_) and positive end-expiratory pressure (PEEP) in mechanical ventilation were gradually escalated. Severe hypoxemia worsened despite aggressive ventilator support with FiO_2_ 1.0 and PEEP over 10 cmH_2_O (Table 1), resulting in a partial pressure of arterial oxygen (PaO_2_) of <70 mmHg (Table 2) and oxygen saturation (SpO_2_) of <90%. Chest radiography revealed worsening pulmonary congestion (Figure 1A), and the demand for inotropics (norepinephrine and vasopressin) increased to the maximum (1 mcg/kg/min and 0.04 units/min, respectively). Echocardiography was performed to rule out cardiogenic pulmonary edema, and no significant functional or structural cardiac abnormalities were observed. These results were consistent with ARDS aggravated by HPS. Since the patient faced life-threatening hypoxemia with conventional ventilation maneuver failure, she was considered an appropriate subject for the initiation of ECMO prior to transplantation.

Thirty hours before the operation, the ECMO team was called to apply VV ECMO. A 21 French (Fr) drainage cannula (DLP^®^, Medtronic Inc., Minneapolis, MN, USA) was placed in the right femoral vein, and a 16 Fr Medtronic return cannula was inserted in the right internal jugular vein. A CAPIOX emergent bypass system (EBS^®^, Terumo Inc., Tokyo, Japan) was applied as the bypass pump. The VV ECMO circulation flow was maintained at 3.5–4.5 L/min with a gas flow of 8–10 L/min at 1900–2200 revolutions per minute (rpm). The FiO_2_ was set to 1.0 for ECMO and 0.8 for the ventilator. Anticoagulation with heparin was not applied before ECMO cannulation due to the patient’s bleeding tendency. The activated clotting time (ACT) was maintained at 250–300 s throughout the cannulation. The patient’s vital signs were well maintained, including SpO_2_ at 95–100%, while waiting for an emergent status 1A liver allocation. Referring to the chest roentgenogram (Figure 1B) and the series of arterial blood gas analysis (ABGA) results (Table 2) after instituting VV ECMO, the patient’s respiratory failure gradually alleviated.

After thirty hours of ECMO administration, the patient was transferred to the operating room to receive DDLT. The total anesthesia time was 460 min, and the total operation time was 425 min. The warm and cold ischemic times of the donated liver were 35 and 320 min, respectively. Perioperative ventilation, ABGA profiles, coagulation profiles, and laboratory test profiles are presented in Table 1, Table 2, Table 3 and Table 4, respectively. Considering the high risk of intraoperative massive bleeding and hemodynamic instability, we decided to suspend CRRT during the operation and resume it, if necessary, after the surgery. The intraoperative ECMO flow was maintained at 3.5–4.0 L/min with a gas flow of 10 L/min at 1900–2200 rpm. The FiO_2_ was set to 1.0 for ECMO, and for the ventilator, it was adjusted based on the serial ABGA follow-up results.

The patient was brought to the operating room already intubated. General anesthesia was induced using desflurane, remifentanil, and vecuronium. For hemodynamic monitoring, right and left radial arterial pressures, left internal jugular venous pressure, cardiac output (CO) monitoring using the FloTrac/Vigileo^TM^ system (Edwards Lifesciences, Irvine, CA, USA) through the right radial artery, and 2–7-megahertz transesophageal echocardiography (TEE) (Philips, X7-2t, Andover, MA, USA) were applied. In our institution, during LT procedures, we typically insert a Swan-Ganz catheter to monitor pulmonary arterial pressure, capillary wedge pressure, and cardiac function, including CO. However, due to the presence of the ECMO cannula, there was no proper cannulation site for a Swan-Ganz catheter, so these monitoring parameters could not be measured. As an alternative, intraoperative TEE was applied for monitoring.

To facilitate bicaval cross clamping during transplantation, the right femoral cannula of the VV ECMO was retracted prior to allograft transplantation. However, this led to inadequate preservation of the ECMO circulation flow, causing hemodynamic instability in the patient. Therefore, the right femoral cannula was placed in its original position, and we decided to proceed with partial clamping, in consideration of the ECMO line located in the vena cava. Consequently, the operation was performed using the piggyback technique while maintaining caval flow.

The patient’s SpO_2_ remained stable, ranging from 95 to 100%, with FiO_2_ of 0.8–1.0 on the ventilator. The tidal volume was regulated at 4–5 mL/kg, the respiratory rate was regulated at 12–16 breaths/min, and PEEP was regulated at 10–15 cmH_2_O (Table 1). Body temperature was maintained within the range 36.0–36.5 °C with the assistance of heating methods via the ECMO line. The total amounts of administered crystalloid, 5% dextrose water, cell saver autotransfusion, leukocyte-depleted red blood cells, fresh frozen plasma, plateletpheresis, and cryoprecipitate during the surgery were 6600 mL, 750 mL, 232 mL, 12 units, 10 units, 1 unit, and 5 units, respectively. Intraoperatively, hematocrit above 27.0% and hemoglobin above 8.0 g/dL were maintained. The mean arterial pressure was appropriately maintained with the adequate administration of norepinephrine infusion (0.025–0.500 mcg/kg/min) without significant hypotensive episodes. No urine was excreted throughout the entire surgery. Hypotension occurred during the reperfusion period but was successfully resuscitated with a total administration of 80 mcg of epinephrine. Post-reperfusion syndrome was not significant, and the patient remained hemodynamically stable after reperfusion. According to the TEE performed after reperfusion, there were no significant changes in cardiac function or volume status. The ejection fraction (EF) was measured using the Modified Simpson’s method during the operation, and the EF measured after reperfusion was 69.89%.

After DDLT, the patient was sedated using a continuous infusion of ketamine and remifentanil, with the administration of vecuronium for neuromuscular blockade. The patient remained hemodynamically stable, allowing for the restart of CRRT independent from ECMO via a parallel system.

**Figure 2:**
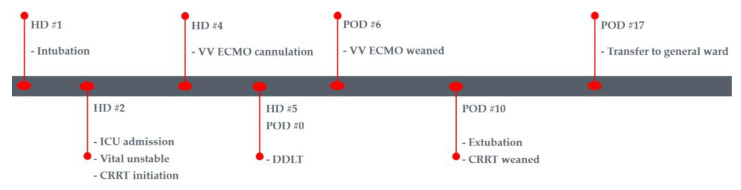
Timeline graph of the patient. HD: hospital day; VV ECMO: veno-venous extracorporeal membrane oxygenation; POD: postoperative day; ICU: intensive care unit; CRRT: continuous renal replacement therapy; DDLT: deceased donor liver transplantation.

From postoperative day (POD) 1 to 3, the inotrope (norepinephrine) was progressively tapered. On PODs 4 and 5, PaO_2_ and chest roentgenogram revealed significant improvement. Additionally, coagulopathy (Table 3) and liver function tests (Table 4) showed improvement, and the graft’s functionality was confirmed through abdominal Doppler ultrasonography. Based on these results, it was concluded that the patient had recovered from acute liver failure. As the patient’s ARDS was highly correlated with acute liver failure, a decision was made to attempt weaning off VV ECMO. To initiate weaning, as the first step, we gradually reduced the bypass flow to 3.0 L/min with rpm reduction, and the FiO_2_ on the ventilator was progressively decreased to 0.4. Close monitoring of vital signs and ABGA follow-ups (Table 2) confirmed that the patient remained in a stable condition. In the second step, the oxygen flow of ECMO was gradually reduced to zero, and the FiO_2_ on the ventilator was increased to 0.7. After this step, the patient continued to remain hemodynamically stable and showed adequate oxygenation in ABGAs (Table 2). Consequently, ECMO was successfully weaned off, and decannulation took place on POD 6. In the transition to conventional ventilator support, the FiO_2_ could be gradually reduced as the patient’s oxygen demand decreased (Table 1). The chest radiograph findings consistently improved (Figure 1C). Extubation was performed on POD 10, and the patient was transferred from the ICU to the general ward on POD 17 as her overall condition showed progressive improvement. The timeline graph (Figure 2) presented below provides a brief overview of the patient’s clinical course. Further evaluation was carried out by the Department of Hepatology to determine the cause of acute liver failure. However, no clear cause could be identified.

## 3. Discussion

The use and advantages of ECMO for ARDS that is unresponsive to conventional ventilator maneuver are well-known in the non-transplantation literature. As respiratory failure occurs relatively frequently in LT [5], there have been a few presented case reports and studies discussing the successful use of intraoperative or postoperative ECMO administration in patients with reversible ARDS [7,8]. However, to the best of our knowledge, cases of ECMO application before transplantation are rarely reported [3,4]. This is likely because LT candidates with ECMO support often have a higher likelihood of mortality while waiting for LT, or many LT candidates do not meet the criteria for ECMO application. Kim et al. [3] reported a case of a patient with hepatitis B cirrhosis complicated by hepatocellular carcinoma and renal failure undergoing combined liver and kidney transplantation. The patient progressed to ARDS with liver allograft dysfunction. The patient successfully underwent re-transplantation with the assistance of VV ECMO. Choi et al. [4] presented a case of a patient with acute-on-chronic liver failure undergoing DDLT. The patient deteriorated due to multiorgan failure including ARDS. With the support of preoperative ECMO, the patient successfully underwent DDLT. As the criteria for LT recipients gradually broaden and the number of operations increases, the need for perioperative ECMO support may rise. It becomes necessary to establish anesthetic management guidelines for perioperative ECMO use in LT. In this discussion, we address the challenging anesthetic management of a patient who received perioperative VV ECMO treatment in the setting of DDLT, which is a rare case.

When utilizing VV ECMO, it is essential to consider the reversibility of pulmonary function as it pertains to the possibility of ECMO weaning. In our case, since the primary cause of the patient’s acute respiratory failure was attributed to acute liver failure, we considered that it could potentially be reversible once the liver failure resolved. Despite further evaluation, the cause of acute liver failure could not be identified, and due to the patient’s gradual deterioration, an emergent LT was decided. We instituted VV ECMO with the expectation that successful weaning from ECMO would be possible after DDLT. In our hospital, the most frequently used standard criterion for initiating ECMO is PaO_2_/FiO_2_ < 100 mmHg with high PEEP (10–20 cmH_2_O) on FiO_2_ > 90% [9]. The patient met this criterion, and considering her aggravating condition, VV ECMO was applied before DDLT.

There are two types of ECMO: VV ECMO, which treats isolated respiratory failure, and veno-arterial ECMO, which supports both cardiac and respiratory failure [1,2,9]. Prior to initiating ECMO, echocardiography was performed to rule out cardiogenic pulmonary edema, and as a result, no structural or functional abnormalities of the heart were observed. Since our patient suffered non-cardiogenic isolated oxygenation failure that did not respond to conventional mechanical ventilation, VV ECMO was implemented. During the surgery, TEE and the FloTrac/Vigileo^TM^ system via the right radial artery were used to evaluate cardiac function, including CO. As there was no suitable cannulation site for a Swan-Ganz catheter due to the ECMO cannula, we administered TEE as an alternative. The TEE method has been verified in terms of measuring CO compared to the thermodilution CO (TDCO) method via Swan-Ganz catheterization [10]. Therefore, we decided to adopt TEE as a substitute for TDCO. Given the patient’s hemodynamic stability and normal cardiac function, including EF, throughout the transplantation, we were able to maintain ECMO in VV mode. We successfully conducted lung-protective ventilation during ECMO treatment (Table 1), which is essential for subsequent lung recovery [2]. The FiO_2_ on the ventilator was modified according to serial ABGA results and maintained between 0.8 and 1.0 during the operation.

One of the challenges in the perioperative management of our patient was a bleeding tendency. During ECMO support, anticoagulation with heparin administration is performed to maintain an ACT of 180 to 220 s or an activated partial thromboplastin time (aPTT) of 40 to 55 s [2,9]. However, in this case, the conventional anticoagulation method could not be employed due to the patient’s severe bleeding tendency. There are no clear guidelines regarding the anticoagulation protocol for LT with ECMO support in cases of a high bleeding tendency. Fina et al. [11] demonstrated that instituting ECMO without anticoagulation was feasible and accomplished effective cardiopulmonary support in patients at high risk of bleeding. We excluded an anticoagulation method on this basis. Although the ACT and aPTT were maintained above the targeted range (Table 3), ECMO treatment was continued throughout the perioperative period without any adverse events, and the bleeding tendency gradually improved after transplantation.

Weaning from ECMO should be conducted with close monitoring of the patient. The function of ECMO should be gradually reduced in stages, and the patient’s tolerance to the decreased level of ECMO support should be evaluated [2]. In our case, the VV ECMO support was successfully weaned off on POD 6 as the patient endured the gradual reduction in ECMO assistance. Despite the relatively short duration of ECMO treatment, the patient’s respiratory function improved significantly after ECMO support and LT, as confirmed by serial chest radiography and ABGAs.

There are several contraindications to LT [12]. Severe pulmonary hypertension or hypoxemia caused by HPS poses an excessive risk to the recipient, which aligns with our patient’s condition. The patient exhibited severe respiratory distress and was clinically unstable despite medical treatment, leading to the conclusion that the patient was contraindicated for LT. However, by implementing preoperative VV ECMO, the ARDS improved, and the patient’s overall condition became more eligible for DDLT. This case indicates that pretransplant VV ECMO can serve as a therapeutic option for LT candidates with acute liver failure combined with reversible respiratory failure to make the patient’s condition more suitable for LT. By employing VV ECMO as a useful bridge therapy for LT, it may be possible to expand the pool of liver failure patients awaiting transplantation. Further case experiences and studies are necessary to establish the appropriate management of perioperative ECMO in LT.

## 4. Conclusions

In conclusion, this case demonstrates that VV ECMO may be a useful therapeutic option not only during the intraoperative and postoperative periods but also during the preoperative period for patients with liver failure combined with reversible respiratory failure.

## Figures and Tables

**Table 1:** Mechanical ventilation profiles.

	FiO_2_	Tidal Volume (mL)	RR (Breaths/min)	I:E Ratio	PEEP (cmH_2_O)	Peak Pressure (cmH_2_O)	Plateu Pressure (cmH_2_O)	Driving Pressure (cmH_2_O)
**Before ECMO**	1.00	400	11	1:2	5	27	26	21
**After ECMO**	0.80	300	14	1:2.5	10	29	28	18
**After induction of anesthesia**	0.85	290	15	1:2	12	30	28	16
**Post-anhepatic, 1 h**	1.00	290	14	1:2.5	16	30	28	12
**After reperfusion**	1.00	260	16	1:2	15	29	27	12
**Post-reperfusion, 1 h**	0.90	295	15	1:3	15	27	24	11
**End of the operation**	0.80	300	14	1:2.5	12	25	23	11
**ECMO weaning, 1st step**	0.40	320	13	1:2	8	20	17	9
**ECMO weaning, 2nd step**	0.70	350	12	1:2	7	19	16	9
**ECMO weaned, 24 h**	0.60	350	15	1:2	5	17	14	9

FiO_2_: fraction of inspired oxygen; RR: respiratory rate; I:E: inspiratory/expiratory; PEEP: positive end-expiratory pressure; ECMO: extracorporeal membrane oxygenation.

**Table 2:** ABGA profiles.

	pH	PaCO_2_ (mmHg)	PaO_2_ (mmHg)	BE (mmol/L)
**Before ECMO**	7.272	33.9	59.1	−15.0
**After ECMO**	7.300	34.3	72.1	−12.4
**After induction of anesthesia**	7.340	24.2	163.4	−10.0
**Post-anhepatic, 1 h**	7.480	20.9	185.6	−13.1
**After reperfusion**	7.370	28.1	105.3	−16.4
**Post-reperfusion, 1 h**	7.400	35.7	196.7	−12.7
**End of the operation**	7.354	31.3	104.5	−6.7
**ECMO weaning, 1st step**	7.358	36.7	96.1	−4.8
**ECMO weaning, 2nd step**	7.351	32.4	95.4	−5.6
**ECMO weaned, 24 h**	7.406	40.2	97.7	−4.7

ABGA: arterial blood gas analysis; pH: hydrogen ion concentration; PaCO_2_: partial pressure of carbon dioxide; PaO_2_: partial pressure of oxygen; BE: base excess; ECMO: extracorporeal membrane oxygenation.

**Table 3:** Coagulation profiles.

	Platelet (1000/mm^3^)	PT (INR)	aPTT (sec)	ACT (sec)
**Before ECMO**	62	4.36	147.2	271
**After ECMO**	77	3.04	100.8	260
**After induction of anesthesia**	59	2.62	85.0	244
**Post-anhepatic, 1 h**	45	2.31	76.2	240
**After reperfusion**	40	>8.00	>156.0	>1000
**Post-reperfusion, 1 h**	33	3.24	145.5	257
**End of the operation**	45	2.10	72.8	202
**ECMO weaning, 1st step**	55	1.70	69.7	157
**ECMO weaning, 2nd step**	67	1.51	51.3	139
**ECMO weaned, 24 h**	85	1.61	38.8	-

PT: prothrombin time; INR: international normalized ratio; aPTT: activated partial thromboplastin time; ACT: activated clotting time; ECMO: extracorporeal membrane oxygenation.

**Table 4:** Laboratory test profiles.

	AST/ALT (U/L)	Total/Direct Bilirubin (mg/dL)	Ammonia (umol/L)	Lactate (mg/dL)	Serum Creatinine (mg/dL)
**Before ECMO**	568/2890	8.9/5.2	149	186.2	1.78
**After ECMO**	526/2070	9.2/5.5	140	182.9	1.41
**After induction of anesthesia**	301/217	11.2/7.0	106	192.8	1.08
**Post-anhepatic, 1 h**	316/238	8.7/6.8	104	167.6	1.69
**After reperfusion**	430/259	11.4/7.1	126	209.4	1.08
**Post-reperfusion, 1 h**	522/302	7.0/4.5	92	150.4	1.00
**End of the operation**	472/257	7.4/3.7	70	120.1	1.06
**ECMO weaning, 1st step**	170/157	3.0/1.4	47	20.4	0.89
**ECMO weaning, 2nd step**	140/121	2.9/1.5	48	24.8	0.78
**ECMO weaned, 24 h**	106/105	2.2/0.8	44	22.4	0.74

AST/ALT: aspartate aminotransferase/alanine aminotransferase; ECMO: extracorporeal membrane oxygenation.

## Data Availability

Not applicable.
